# Combined integrated protocol/basket trial design for a first-in-human trial

**DOI:** 10.1186/s13023-016-0494-z

**Published:** 2016-10-04

**Authors:** Ulla Derhaschnig, Jim Gilbert, Ulrich Jäger, Georg Böhmig, Georg Stingl, Bernd Jilma

**Affiliations:** 1 Department of Clinical Pharmacology, Medical University of Vienna, Währinger Gürtel 18-20, 1090 Vienna, Austria; 2 Department of Emergency Medicine, Medical University of Vienna, Vienna, Austria; 3 True North Therapeutics, San Francisco, CA USA; 4 Department of Internal Medicine I, Division of Haematology, Medical University of Vienna, Vienna, Austria; 5 Department of Internal Medicine III, Division of Nephrology and Dialysis, Medical University of Vienna, Vienna, Austria; 6 Department of Dermatology, Division of Immunology, Allergy and Infectious Diseases, Medical University of Vienna, Vienna, Austria

**Keywords:** First-in-human trial, Basket design, Integrated protocol design, Orphan disease, Complement pathway

## Abstract

**Background:**

Innovative trial designs are sought to streamline drug development in rare diseases. Basket- and integrated protocol designs are two of these new strategies and have been applied in a handful oncologic trials. We have taken the concept outside the realm of oncology and report about a first-in-human integrated protocol design that facilitates the transition from phase Ia in healthy volunteers to phase Ib in patients with rare complement-mediated disorders driven by the classical pathway.

**Results:**

We have been conducting a prospective, double-blind, randomized, placebo-controlled first-in-human study with TNT009, which is a humanized monoclonal antibody directed against the C1s subunit of human complement component C1. The trial consisted of three subparts, including normal healthy volunteers (part one and two) and a single cohort of patients in part three. Patients suffered from various complement-mediated diseases sharing the same pathophysiological mechanism, i.e. bullous pemphigoid, antibody-mediated rejection of organ transplants, cold agglutinin disease and warm autoimmune hemolytic anemia. Primary objective of the trial has been to evaluate the safety and tolerability of TNT009 in humans.

**Conclusions:**

This trial provides probably the first example that basket trials may not be limited to single genetic aberrations, which is overly restrictive, but our trial design demonstrates that pathway specificity is a viable paradigm for defining baskets. This will hopefully serve as a role model that could benefit other innovative drug development programs targeting rare diseases.

## Background

The definition and implementation of innovative trials to accelerate access to efficacious and safe medicines is of major interest to patients, industry and regulators [1]. On a European level, the Framework Programme 7 has been supporting various multinational collaborative efforts to develop new methodologies for making clinical trials in rare diseases more efficient [2–4]. These trial design strategies comprise, for example, integrated protocol designs and another related emerging concept, the basket trial design. Basket trials enroll patients with multiple diseases and one (or more) drug targets in cohorts or groups within one trial [5]. Thereby it is possible to identify a potentially favorable response to targeted therapy with a small number of patients [6]. Thus, basket designs have been successfully applied in a handful oncologic trials dealing with rare cancers [7].

We have taken the concept of basket trial design outside the realm of Oncology and have now developed a first-in-human integrated protocol design that facilitates the transition from Phase Ia in normal healthy volunteers (NHVs) to Phase Ib in patients with various complement-mediated, orphan diseases in hematology, transplant medicine and dermatology that share the same molecular target for drug therapy.

## Methods

### Protocol design

The trial protocol was approved by the National Competent Authority and the Ethics Committee of the Medical University of Vienna and is registered at ClinicalTrials.gov (NCT 02502903) and EUDRACT (EUDRA-CT 2014-003881-26).

We have been conducting a prospective, double-blind, randomized, placebo-controlled first-in-human study with TNT009 which has recently received Orphan Drug designation by the European Medicines Agency for the treatment of autoimmune hemolytic anemia. TNT009 is a humanized monoclonal antibody directed against the C1s subunit of human complement component C1 [8]. TNT009 is intended for treatment of the subset of complement-mediated disorders that are driven by the classical pathway (CP) [9]. The clinical development plan is currently focused on different autoimmune diseases and rejection of allografts. Antibody-mediated rejection triggered by preformed or de novo donor-specific antibodies is an important cause of graft dysfunction and loss [10]. There are several lines of experimental and clinical evidence that activation of complement via the CP activation substantially contributes to alloantibody-mediated tissue damage [11]. Warm autoimmune hemolytic anemia has an estimated prevalence of 2.3/10000. It can often be managed with corticosteroids, splenectomy, or off-label rituximab [12], which methods, however, are associated with a relevant percentage of treatment failures, relapses, and potentially severe adverse effects. Cold agglutinin disease is an autoimmune hemolytic anemia with an estimated prevalence of 0.3/10000. It is caused by IgM-induced CP activation which is typically exacerbated by exposure to cold environmental temperatures or viral infections [13], for which no treatment is authorized. Bullous pemphigoid is a serious autoimmune blistering skin disease with a prevalence of 1/40000. It has a poor long-term prognosis associated with serious co-morbidity and increased mortality [14]. While bullous pemphigoid can usually be successfully treated by steroids, their long term use commonly leads to severe adverse events in the elderly population.

Although the above seems to represent a clinical trial population with a diverse set of clinical diagnoses, in fact they are united by a common mechanism of disease matched to the mechanism of action of TNT009. Preceding in vitro studies where TNT009 was spiked into blood samples drawn from patients confirmed the strength of this linkage of pathobiology and pharmacology for each of these conditions.

The approved trial protocol has three sub-parts, which have been conducted sequentially: part A, a single ascending dose trial in NHVs, part B, a multiple ascending dose trial in NHVs, and part C, a multiple dose trial in patients with various complement-mediated disorders as described above. The route of administration, dose, and dosing interval for TNT009 were based upon the corresponding non-clinical safety, pharmacokinetic, and pharmacodynamic observations after administration to non-human primates together with the findings from human in vitro pharmacology studies (No Observed Adverse Effect Level (NOAEL) of 100 mg/kg, first-in-human-dose, i.e. 1/300th of the NOAEL, 0.3 mg/kg, threshold plasma level 10 μg/mL).

Part A has been conducted according to an ascending dose cohort paradigm, in which a unique cohort of NHVs has been treated at each single dose level. There have been 7 cohorts of NHVs. NHVs in each cohort have been randomly assigned to receive TNT009 or placebo. In each cohort at least one participant has been treated with placebo in order to preserve the blinded treatment and to serve as control for the safety analysis.

The first two cohorts have consisted of 4 subjects each, 3 given TNT009 (0.3 or 1 mg/kg) by intravenous (IV) infusion, and 1 given placebo. The remaining 5 cohorts have consisted of 8 subjects each, 6 given TNT009 by IV infusion (3, 10, 30, 60, or 100 mg/kg) and 2 given placebo.

Not all subjects in a given cohort in part A have been dosed on the same day: a sentinel pair (1 TNT009, 1 placebo) has been dosed on the first day, then staggered groups of 2–3 per day thereafter. The sentinel pair of subjects was incorporated into the design in the interest of safety, i.e. to avoid exposing multiple subjects to any given dose level before making an initial assessment of early adverse events in one, and the single member of this pair was treated with placebo in order to preserve the blinded treatment.

There were no adverse or toxic effects seen in preclinical testing. So the probability for dose escalation based on preclinical results was expected to be > 90 % for this investigational monoclonal antibody. However, no formal calculation of observed/expected dose-limiting toxicity rates with respect to clinical decision-making was involved.

Part B also has been conducted according to an ascending dose cohort paradigm. There have been 2 cohorts of NHVs, each consisting of 8 subjects. These cohorts have been given 4 weekly IV doses of TNT009 or placebo (6:2 active:placebo) at a dose level previously administered to NHVs in the first part (30 or 60 mg/kg). Again, not all subjects in the cohorts have been dosed on the same day: a sentinel pair (1 TNT009, 1 placebo) has been dosed on the first day, then staggered groups of 2–3 per day have been dosed thereafter.

Part C is being conducted in a single cohort of patients. All patients have received a single IV test dose of 10 mg/kg followed by 4 weekly doses of 60 mg/kg (after 1–4 days). As expected for a trial in patients, patients have not been started on the same day, because recruitment of these rare patients with orphan diseases has been a gradual process spanning a number of weeks (Table 1).

**Table 1:**
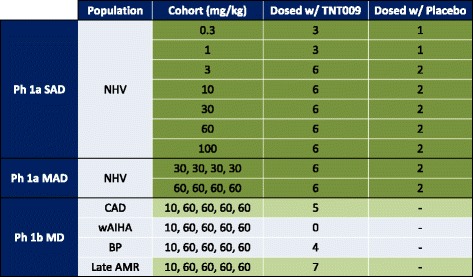
Trial design

*SAD* single ascending dose, *MAD* multiple ascending dose, *MD* multiple dose, *NHV* normal healthy volunteers, *CAD* cold agglutinin disease, *wAIHA* warm agglutinin hemolytic anemia, *BP* bullous pemphigoid, *AMR* antibody mediated rejection The number of patients will increase with further recruitment

The primary endpoint of the trial has been the overall safety and tolerability of TNT009 both in healthy volunteers as well as patients suffering from different target organ diseases. This has been assessed in terms of serious, drug-related adverse events, premature termination due to drug-related adverse events and patterns of all drug-related adverse events and (or) clinical and laboratory abnormalities suggestive of one or more specific target organs for toxicity of TNT009. Secondary endpoints comprised pharmacokinetic and pharmacodynamic endpoints (i.e., complement system parameters, coagulation system parameters and different disease related parameters) to determine the optimal dosage regimen suitable for evaluation in phase II studies, and to prioritize the therapeutic indications for further clinical development.

No classical sample size estimation was performed. However, a sample size of either 4 subjects (3 TNT009, 1 placebo) or 8 subjects (6 TNT009, 2 placebo) per dosing cohort in part A, and 8 subjects (6 TNT009, 2 placebo) per dosing cohort in part B is in compliance with the standard 3 + 3 Phase 1 design [15]. In part C a sample size of, for example, 10 patients for each disease group studied allows for the estimation of an event rate of 30 % with precision of ± 28.4 %. Thus, the target enrollment number of patients for each of the specific complement-mediated disease entities has been between 5 and 20 patients.

For statistical analyses data have been summarized by dose cohort and, where appropriate, by visit. Descriptive statistics will be provided for continuous variables. Frequency counts and percentages will be presented for categorical variables.

A two-sided significance level of 5 % has been applied and two-sided 95 % confidence intervals have been calculated.

## Results and discussion

Basket trials have been developed as an efficient way of screening experimental therapeutics across multiple patient populations in early-phase drug development. They offer the possibility to include multiple molecularly defined subpopulations in one cohesive design to evaluate the targeted therapy in question [7]. So far, however, the number of published trials is very limited. We reasoned that such a study design would not only be suitable for different rare diseases sharing a common genetic aberration but also acquired disease sharing a similar pathophysiological mechanism that can be targeted by a monoclonal antibody against a single target. In our trial this is the subset of complement-mediated disorders that are driven by the CP. There is a considerable gain in efficiency by running multiple cohorts in this way, because conducting a stand-alone trial within each cohort separately would be more resource-, labor- and time-intensive [7]. In order to maximize efficiency, we went further and combined the objectives traditionally addressed in separate trials into a single study, applying an integrated protocol design for the transition of Phase Ia to Phase Ib within one trial. The integration of these two components under one protocol, at one study site, under one Principal Investigator was deemed most appropriate because this enabled the most comprehensive and consistent evaluation of the safety, pharmacokinetics (PK), and pharmacodynamics (PD) of TNT009. Certainly, the primary and secondary endpoints in this trial are analyses that encompass all subjects- whether NHVs or patients- enrolled, profiling general human safety and characterization of PK and PD. Assessment of homogeneity, or comparison of aspects of heterogeneity within NHVs with single or multiple dosing, or between NHVs and patients with multiple dosing, are highly relevant analyses that will inform subsequent clinical development. It is conceivable that target-related differences in PD, or disease-related differences in PK exist for a novel biotherapeutic agent, and the basket design is extraordinarily efficient at uncovering such differences.

Of the four diseases included in our study design, each needs to be considered individually with respect to learning that informs subsequent clinical development. The primary endpoint was defined as an assessment of safety and overall tolerability for all participants including volunteers and different patient cohorts. Similarly, the key secondary endpoints were defined as assessment of PK and PD of TNT009 for all patient strata and healthy volunteers. There is general agreement that safety, PK, and PD in the target population comprise the *sine qua non* deliverables of a Phase Ib clinical trial. Thereafter, in the hierarchy of value some disease strata emerge as potentially more informative than others with regard to the potential to achieve biomarker-based proof of mechanism or, better yet, clinical proof of concept (POC). In the stratum of Cold Agglutinin Disease patients it was anticipated that cessation of hemolysis and correction of anemia could potentially be accessible endpoints within the scope of the trial’s size and duration, provided that it was possible to enroll patients with highly active disease and that the effect of TNT009 was rapid and robust. On the other hand, in the stratum of patients with antibody-mediated rejection it was hoped that biomarker-based proof of mechanism might be achieved by means of a gene expression array (molecular microscopy). Since the path to Phase II in such patients must traverse a Phase Ib safety and PK/PD characterization in the target population, then the hope for biomarker-based proof of mechanism represented “upside potential” added in to our Phase Ib design.

To generalize from our experience with this specific pathway in these specific rare diseases, we believe that the potential for biomarker-based proof of mechanism or clinical POC is a relevant value-added; it could encourage biopharma sponsors to consider profiling novel biotherapeutic agents more broadly than they otherwise would early in clinical development. Does this strategy provide a universal “rule-in” or “rule-out” logic to such decision-making? No, and indeed absence-of-evidence with respect to early proof of mechanism or POC is not evidence-of-absence. However, it does offer a pragmatic approach to prioritization of target indications to pursue for larger-scale clinical development, in that most sponsors will choose to invest first in an indication where proof of mechanism or POC has already been obtained. In this sense our application of the basket trial paradigm allows sponsors to “play the winner” and put the disease with proof of mechanism or POC on the fast track for full development at the earliest possible point in time.

However, new clinical trial designs such as this are not without their limitations. In order for basket trials to succeed some key preconditions must be fulfilled [6, 7], the first of which being a strong scientific rationale for the molecular marker-drug pairing [7] as in our case. Preclinical tests revealed that TNT009 binds with high affinity and specificity to C1s of humans in vitro and non-human primates in vivo [8] and exhibited disease-relevant inhibitory activity against the CP in a variety of in vitro disease models. Pharmacology studies evaluating the effect of TNT009 spiked into blood samples drawn from patients with the diseases under investigation, confirmed the strength of this linkage of pathobiology and pharmacology for each of these diseases. Furthermore, reliable assays for the marker of interest, complement system parameters and disease-related biomarkers, exist and are well established.

An integrated trial protocol must meet additional design requirements, as outlined by the Clinical Trial Facilitation Group (CTFG) of the Heads of Medical Agencies [16]; for example, a pre-defined maximal dose and clear stopping rules for dose escalation are necessary. For our trial we established specific dose escalation criteria and timing to allow progression between dose cohorts and implemented a Data Safety Monitoring Board, consisting of a statistician, dermatologist, nephrologist, hematologist and intensivist, for safety monitoring.

Protocol modifications to the ongoing study have been made in amendments and independent approval for substantial amendments has been obtained from the Ethics Committee. Data from literature suggest that it is not uncommon to have three to five protocol amendments after the initiation of a clinical trial [17]. The adaptive approach might result in a higher number of substantial amendments, which theoretically might cause operational bias that would be relevant for pivotal clinical trials. However, the currently running Phase Ia/Ib trial is focused on safety and POC and not a pivotal Phase III trial.

Statistical considerations will continue to play a central role in basket and integrated trial designs [18, 19]. To avoid pitfalls, we established a statistical analysis plan with a detailed protocol. For part A and B, sample size was calculated using an adapted 3 + 3 phase I design for dose escalation [15]. In part C the sample size was determined by the likely number of available patients with those orphan diseases. Further justification was given by calculation of the detectable adverse event rates. This is in line with recently published recommendations for Phase I studies in Oncology or rare diseases [17, 19]. Finally, as mentioned above, we applied an independent data safety monitoring board to ensure quality, validity and integrity of the collected data [15, 17, 18].

## Conclusion

In conclusion, we believe that based on a strong scientific rationale and in accordance with the guidelines published [15, 16], the application of new innovative trial designs in a non-oncologic environment will combine safety for the patients with increased operational efficiency. This may streamline the development of new compounds in rare diseases, perhaps especially in Europe, where the regulatory climate is conducive to the conduct of such trials.

## Abbreviations

CP, classical pathway; CTFG, Clinical Trial Facilitation Group; NHV, Normal healthy volunteer; PD, pharmacodynamics; PK, pharmacokinetics; POC, proof of concept
